# Anti-hyperuricemia effect of hesperetin is mediated by inhibiting the activity of xanthine oxidase and promoting excretion of uric acid

**DOI:** 10.3389/fphar.2023.1128699

**Published:** 2023-04-12

**Authors:** Meng-Fei An, Chang Shen, Shao-Shi Zhang, Ming-Yue Wang, Ze-Rui Sun, Mao-Si Fan, Li-Juan Zhang, Yun-Li Zhao, Jun Sheng, Xuan-Jun Wang

**Affiliations:** ^1^ Key Laboratory of Pu-erh Tea Science, Ministry of Education, Yunnan Agricultural University, Kunming, China; ^2^ College of Science, Yunnan Agricultural University, Kunming, China; ^3^ College of Food Science and Technology, Yunnan Agricultural University, Kunming, China; ^4^ School of Basic Medicine, Yunnan University of Chinese Medicine, Kunming, China; ^5^ Key Laboratory of Medicinal Chemistry for Natural Resource, Ministry of Education and Yunnan Province, Yunnan Characteristic Plant Extraction Laboratory, Yunnan Provincial Center for Research and Development of Natural Products, School of Pharmacy, School of Chemical Science and Technology, Yunnan University, Kunming, China; ^6^ State Key Laboratory for Conservation and Utilization of Bio-Resources in Yunnan, Kunming, China

**Keywords:** hesperetin, hyperuricemia, xanthine oxidase, NLRP3, excretion

## Abstract

Hesperetin is a natural flavonoid with many biological activities. In view of hyperuricemia treatment, the effects of hesperetin *in vivo* and *in vitro*, and the underlying mechanisms, were explored. Hyperuricemia models induced by yeast extract (YE) or potassium oxonate (PO) in mice were created, as were models based on hypoxanthine and xanthine oxidase (XOD) in L-O2 cells and sodium urate in HEK293T cells. Serum level of uric acid (UA), creatinine (CRE), and urea nitrogen (BUN) were reduced significantly after hesperetin treatment *in vivo*. Hesperetin provided hepatoprotective effects and inhibited xanthine oxidase activity markedly, altered the level of malondialdehyde (MDA), glutathione peroxidase (GSH-PX) and catalase (CAT), downregulated the XOD protein expression, toll-like receptor (TLR)4, nucleotide binding oligomerization domain-like receptor family pyrin domain-containing 3 (NLRP3) inflammasome, interleukin-18 (IL-18), upregulated forkhead box O3a (FOXO3a), manganese superoxide dismutase (MnSOD) in a uric acid-synthesis model in mice. Protein expression of organic anion transporter 1 (OAT1), OAT3, organic cationic transporter 1 (OCT1), and OCT2 was upregulated by hesperetin intervention in a uric acid excretion model in mice. Our results proposal that hesperetin exerts a uric acid-lowering effect through inhibiting xanthine oxidase activity and protein expression, intervening in the TLR4-NLRP3 inflammasome signaling pathway, and up-regulating expression of FOXO3a, MnSOD, OAT1, OAT3, OCT1, and OCT2 proteins. Thus, hesperetin could be a promising therapeutic agent against hyperuricemia.

## Graphical Abstract

**Figure:**
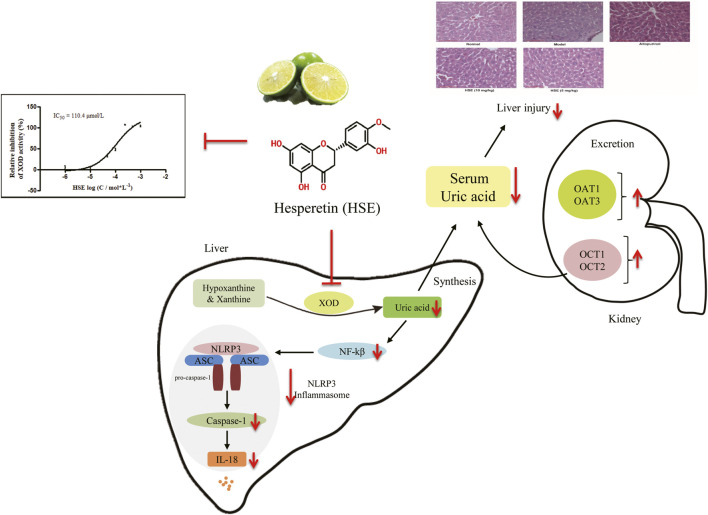
Both *in vivo* and *in vitro* experiments suggested that HSE may effectively lower uric acid. The mechanism might be the inhibition of XOD activity, down-regulation of TLR4-NLRP3 inflammasome and up-regulation expression of OAT1, OAT3, OCT1, OCT2 proteins.

## 1 Introduction

Hyperuricemia is a metabolic disorder caused by an imbalance in the production and excretion of uric acid (UA), and is considered to be a risk factor for gout, obesity, chronic kidney disease, and diabetes mellitus (Bakan et al., 2015; Gromadzinski et al., 2015; Ali et al., 2018). Studies have demonstrated that lowering the prevalence of hyperuricemia can reduce the risk of other metabolic diseases (Borghi et al., 2020; Huang et al., 2020; Xie et al., 2020; Yen et al., 2020).

According to the mechanism of action, the current prevention and treatment strategy against hyperuricemia is reducing uric acid production and/or enhancing urate excretion to lower the levels of serum uric acid. Xanthine oxidase (XOD) catalyzes the oxidation of hypoxanthine or xanthine to uric acid in the liver (Chen et al., 2016; Qin et al., 2018), which is considered to be a promising therapeutic target for hyperuricemia caused by overproduction of uric acid (Richette and Bardin, 2010). Allopurinol and febuxostat inhibit uric acid production by ablating xanthine oxidase activity, but they are suitable only for patients with early hyperuricemia and not for people with renal disease/insufficiency (Kai et al., 2013). Considerable evidence supports that uric acid transporters are critical for uric acid metabolism in the kidneys (Jalal et al., 2013; Tan et al., 2016). Organic cationic transporters (OCTs) and organic anion transporters (OATs) have been reported to mainly promote urate excretion (Wang et al., 2019). Benzbromarone and probenecid lower the level of uric acid by regulating expression of uric acid transporters in the kidney, but their use is associated with renal damage and hepatotoxicity (Zhang et al., 2006; Theilmann and Ormiston, 2019). Some dual-target drugs can interfere with the synthesis and metabolism of uric acid, such as RLBN1001 (Chen, 2017). Clinical application of drugs for hyperuricemia treatment is limited due to the high prevalence of adverse reactions. Bioactive compounds of plant origin have strong potential to treat a variety of disease, including hyperuricemia (Mehmood et al., 2019a; Mehmood et al., 2019b; Serrano et al., 2020).

For now, the hyperuricemia cell model has become a new research direction due to its high screening efficiency. Uric acid is synthesized in the liver, and the metabolic process is roughly adenine ribonucleotide-adenosine-inosine-hypoxanthine-xanthine-uric acid. Increasing uric acid or its precursors can lead to increased blood uric acid levels (Adachi et al., 2017). Previous Studies reported that L-O2 cells can be successfully induced as a hyperuricemia cell model under the co-induction of hypoxanthine (the direct precursor of uric acid production) and xanthine oxidase (An et al., 2021; Sun et al., 2021). Additionally, uric acid-induced renal epithelial cells (HEK293T cells or HK-2 cells) model are commonly used to study the effects of compounds on uric acid uptake or transport (Bao et al., 2019; Feng et al., 2019). Therefore, both models were selected to evaluate the uric acid-lowering effect of hesperetin.

The NLRP3 inflammasome, as part of the innate immune system, recognizes numerous pathogen types and has attracted the attention of researchers. Studies demonstrate that uric acid promote activation of the nucleotide binding oligomerization domain-like receptor family pyrin domain-containing 3 (NLRP3) inflammasome, and lead to release of the downstream pro-inflammatory factor interleukin (IL)-18 (Ives et al., 2015; Chen et al., 2018). Thereby, inhibition of the NLRP3 inflammasome is a practicable strategy to alleviate side effects of hyperuricemia.

**FIGURE 1:**
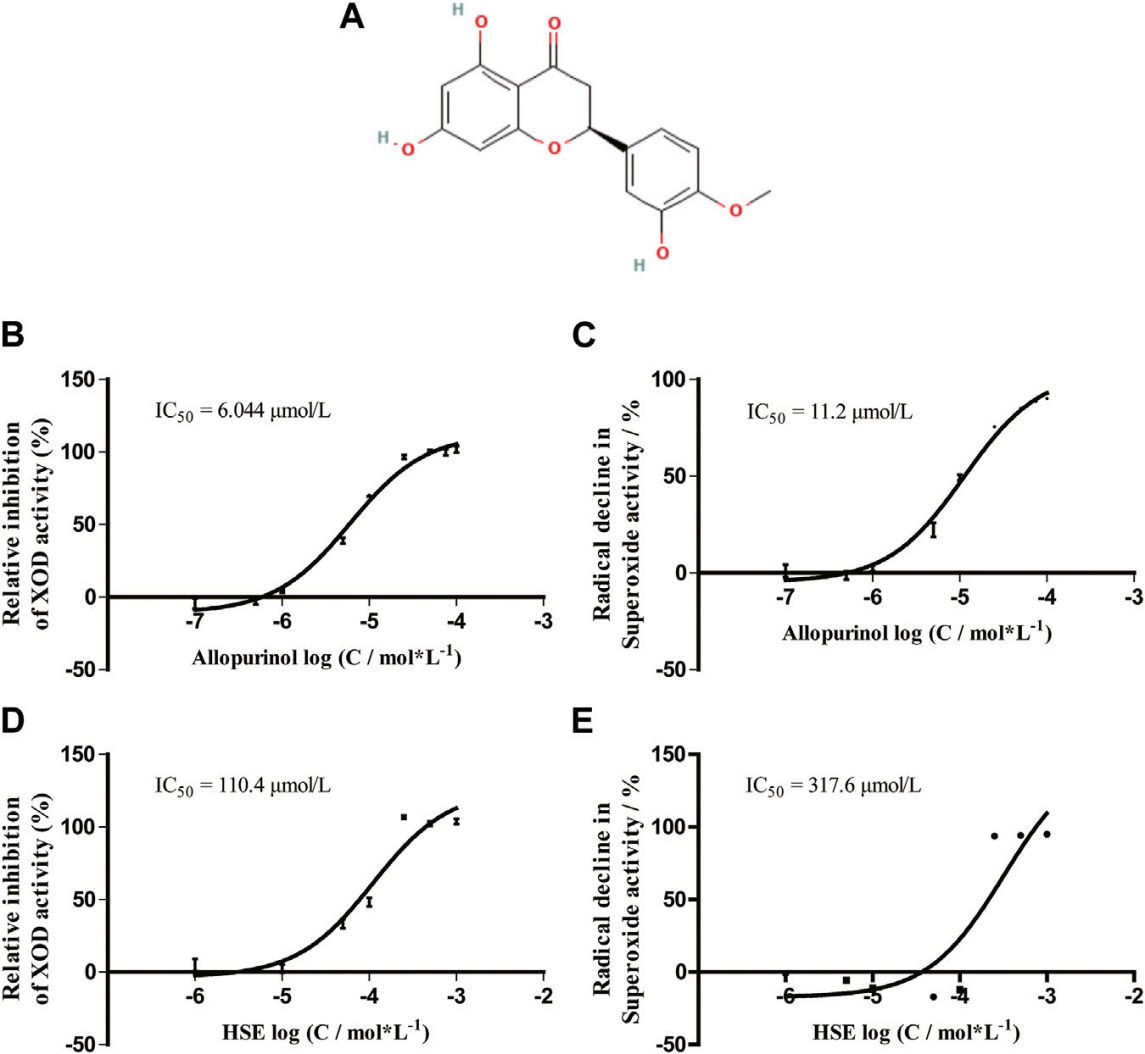
Chemical structure of hesperetin (HSE) **(A)** and its dose-response curve on xanthine oxidase (XOD) activity **(D)** and scavenging of superoxide anions **(E)** *in vitro*. Data are the mean ± SEM (n = 3) and representative of three independent experiments. The effect of allopurinol on XOD activity **(B)** and scavenging of superoxide anions **(C)** are shown. HSE, hesperetin; XOD, xanthine oxidase.

Hesperetin (Figure 1A) is a flavonoid derived mainly from the citrus fruit of the Rutaceae family, such as grapes, lemons, tangerines, and oranges (Garg et al., 2001; Wolfram et al., 2016; Chen et al., 2017). Hesperetin has various biological activities, such as anti-inflammatory (Wang et al., 2016; Bai et al., 2017; Lin et al., 2020), antioxidant (Gong et al., 2017), anti-fibrosis (Wang B. et al., 2017; Wang H. W. et al., 2017) and neuroprotective (Choi and Ahn, 2008; Shagirtha et al., 2017; Kheradmand et al., 2018) activities. Studies have shown that diets containing hesperetin provide cardioprotective effects, and reduce the morbidity and mortality of coronary heart disease by lowering plasma levels of low-density lipoprotein-cholesterol (Bawazeer et al., 2016). Drinking orange juice can help reduce hyperuricemia (Haidari et al., 2012), and it has been demonstrated that hesperetin has an inhibitory effect on xanthine oxidase (Haidari et al., 2009). Though, the mechanism of action of hesperetin on uric acid metabolism is not known. The aim of this study was to investigate the effect of hesperetin on uric acid metabolism and to explore its mechanism of action *in vitro* and *in vivo*.

## 2 Materials and methods

### 2.1 Reagents

Hesperetin (95%–99%; Chemical Abstracts Service (CAS) number: 520-33–2) was purchased from Chengdu Purifa Technology Development (Chengdu, China). Xanthine oxidase (XOD), allopurinol, uric acid, sodium urate (SU) (BCCB4889); xanthine (CAS-69-89–6), and yeast extract (YE) (2340951-02) were obtained from Sigma-Aldrich (Saint Louis, MO, United States). Potassium oxonate (PO) (01045378) was from Damas-beta (Shanghai, China). Hypoxanthine (H108384) was purchased from Aladdin (Shanghai, China). XOD kit and UA kits were procured from Nanjing Jiancheng Bioengineering Institute (Nanjing, China). 3-(4,5-dimethylthiazol-2-yl)-2,5-diphenyltetrazolium bromide (MTT) was from Solarbio (Beijing, China). WST-1 (W201) was purchased from Dongren Chemicals (Shanghai, China). Anti-XOD antibody [EPR4605] (ab109235), anti-NLRP3 antibody [EPR20425] (ab210491), and anti-IL-18 antibody [EPR19956] raised against mice were purchased from Abcam (Cambridge, UK). Anti-OAT1 (1F2): sc-293323, anti-OAT3 (3C11): sc-293264, anti-OCT1 (12F11): sc-8024 and toll-like receptor (TLR)4: sc-293072 antibodies raised against mice were purchased from Santa Cruz Biotechnology (Santa Cruz, CA, United States). Anti-OCT2 (H-3): sc-377476 and NF-κB p65 (C22B4) antibodies were obtained from Cell Signaling Technology (Beverly, MA, United States). Anti-β-tubulin (66240-1-Ig) was purchased from Proteintech (Rosemont, IL, United States). Horseradish peroxidase (HRP)-conjugated goat anti-mouse IgG or anti-rabbit IgG were obtained from R&D Systems (Minneapolis, MN, United States).

### 2.2 Animals

Animal studies (animal ethics approval code number: YUAN2019LLWYH003-2) were undertaken according to recommendations set in Guide for the Care and Use of Laboratory Animals [United States National Institutes of Health (Bethesda, MD, United States)]. Male specific pathogen-free (SPF) Institute of Cancer Research (ICR) mice (20–22 g) were purchased from Kunming Medical University (license number: SCXK 2015-0004). In order to adapt to the environment, animals were housed in an SPF-grade laboratory (license number: SYXK 2018-0005) with a 12-h light-dark cycle (20°C–25°C, 60% ± 10% humidity) in the animal house. Free food and water were supplied for 1 week before performing any procedure.

### 2.3 Assay of xanthine oxidase activity *in vitro*

Uric acid (which has maximum optical density at 295 nm (OD_295_)) works as an indicator of xanthine oxidase activity. According to the literature (Qin et al., 2018), a 100-μL reaction system comprising xanthine oxidase (40 U/L), xanthine (250 mmol/L), WST-1 (100 μmol/L), and test compounds at various concentrations in reaction buffer (0.1 mol/L sodium paraphosphate, 0.3 mmol/L ethylenediamine tetraacetic acid, pH 8.3). The OD_295_ of the reaction system was monitored dynamically for 20 min at 25°C with a microplate reader (SpectraMax M3; Molecular Devices, Sunnyvale, CA, United States). The slope of the OD_295_-time curve reflects xanthine oxidase activity. The slope of the OD_450_-time curve represents the O^2-^ level. The dose-response curve was obtained and the half-maximal inhibitory concentration (IC_50_) value of each compound was calculated.

### 2.4 Dose regimen

Hesperetin and allopurinol were prepared in saline solution containing 1% Tween-80 immediately before the use to obtain a uniform suspension and the administrated volume was 10 mL/kg. Previous studies have shown that intraperitoneal administration of 20 mg/kg HSE can prevent ototoxicity by increasing antioxidant enzymes and reducing oxidation parameters, as well as prevent apoptosis caused by cochlear implant cell proliferation (Kara et al., 2016), and the experimental dose adjustment in this study is based on this.

### 2.5 Uric acid-lowering effect of hesperetin on yeast extract-induced hyperuricemia in mice

**FIGURE 2:**
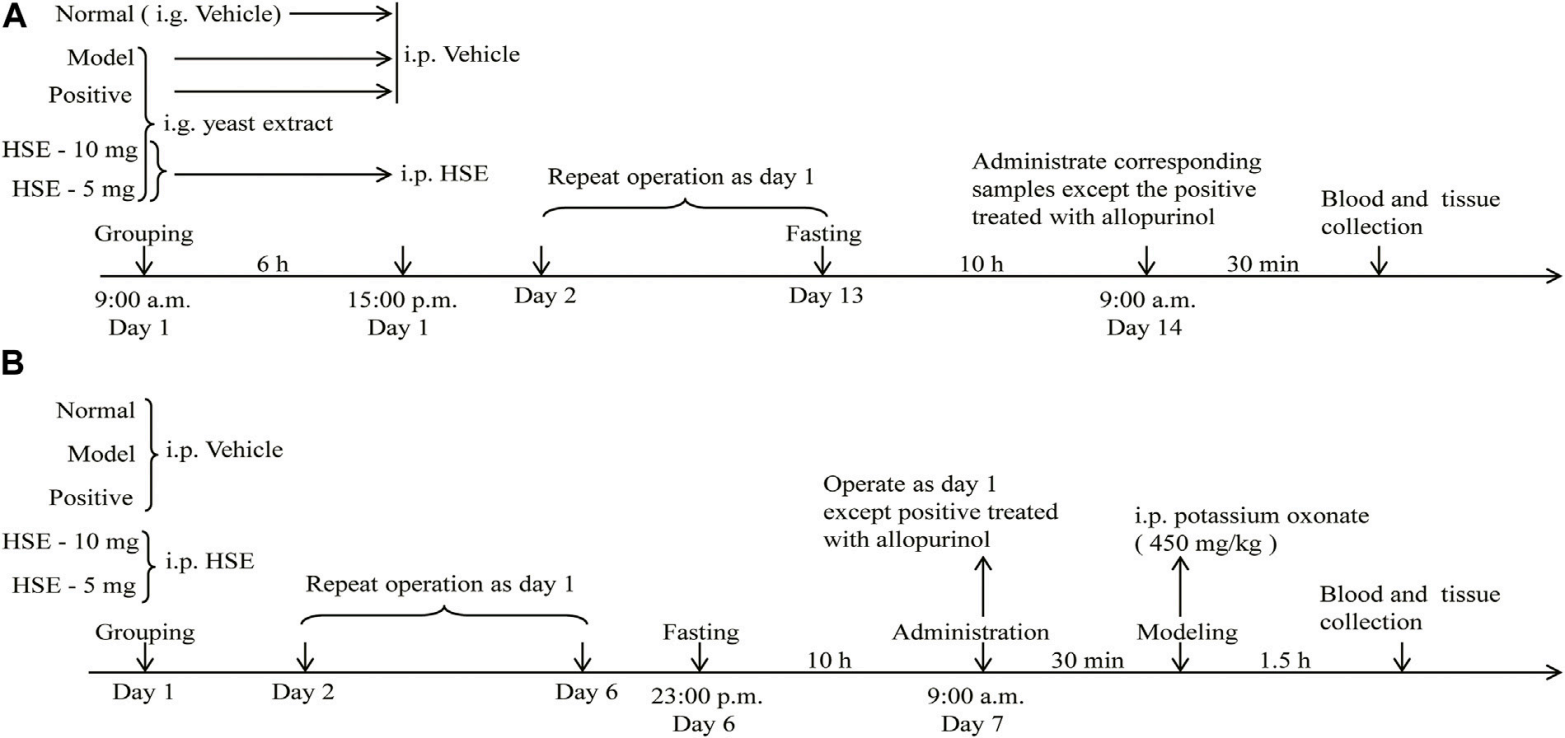
The flowchart of animal experiments induced by yeast extract **(A)** and potassium oxonate **(B)** hyperuricemia. i. g., intragastric administration; i. p., intraperitoneal injection; HSE, Hesperetin; vehicle, saline solution contain 1% Tween-80.

Refer to the previous methods (An et al., 2021) and dosage regimens (He et al., 2020), mice were randomly assigned into five groups: normal; model; positive-treated (allopurinol, 10 mg/kg); hesperetin (10 mg/kg)-treated; hesperetin (5 mg/kg)-treated. Mice in the normal group underwent intragastric administration with saline solution contain 1% Tween-80 (vehicle) at 10 mL/kg for successive 13 days. Instead, mice in other groups were orally administrated with yeast extract (15 g/kg). After 6 h, hesperetin group was given the corresponding dose (10 and 5 mg/kg), the positive-treated and the normal group accepted the administration of same volume of vehicle. After 13 days, mice were fasted for 10 h, and then the positive allopurinol was intragastrically administered once according to the report of Niu et al. (2018), and the other groups were administered by intraperitoneal injection of the corresponding articles. After 30 min, blood was collected *via* the orbital vein. Serum samples were obtained by centrifuging whole-blood samples at 4,000 × *g* for 10 min at 4°C. Serum levels of uric acid, creatinine, and urea nitrogen were determined using an automatic biochemistry analyzer (AU680; Beckman Coulter, Fullerton, CA, United States). Hepatic xanthine oxidase activity was measured using the xanthine oxidase kit. The study flowchart was shown in Figure 2A.

### 2.6 Evaluation of factors related to oxidase stress in livers induced by yeast extract

The levels of malondialdehyde (MDA), glutathione peroxidase (GSH-PX), catalase (CAT) activity in the livers of mice induced by yeast extract were determined using kits (Nanjing Jiancheng Bioengineering Institute, Nanjing, China).

### 2.7 Histopathology in yeast extract-induced hyperuricemia

Left upper-lobe liver tissues were embedded in paraffin, cut into 5 μm thick slices, and stained with hematoxylin and eosin staining. We evaluated the general morphology under light microscopy, as reported previously (Xiong et al., 2017).

### 2.8 Uric acid-lowering effect of hesperetin on potassium oxonate-induced hyperuricemia in mice

Mice were randomly assigned into five groups: normal; model; allopurinol (10 mg/kg)-treated; hesperetin (10 mg/kg)-treated; hesperetin (5 mg/kg)-treated. Mice in the hesperetin group were injected once a day for consecutive 6 days. The normal, model group and positive group received the vehicle in the same manner. On the seventh day, HSE group mice, fasted for 10 h, were injected subcutaneously to avoid mutual interference with the modeling reagent. The positive control received one gavage of allopurinol (10 mg/kg). 30 min after administration, all groups were administered potassium oxonate (450 mg/kg, i. p.) except that the normal control group was injected with an equal volume of vehicle. The study flowchart was shown in Figure 2B.

Blood was drawn from the inner canthus after modeling for 1.5 h. After killing, the kidney was frozen in liquid nitrogen and stored at −80°C for later use. Serum samples were obtained by centrifuging blood samples at 4,000 × *g* for 10 min 4°C. Serum levels of uric acid, creatinine, and urea nitrogen were determined using an automatic biochemistry analyzer (AU680; Beckman Coulter).

### 2.9 Cell culture and MTT assay

L-O2 cells purchased from Shanghai Meixuan Biotechnology (Shanghai, China) and HEK293T cells (American Type Culture Collection, Manassas, VA, United States) were cultured in Dulbecco’s modified Eagle’s medium (DMEM) supplemented with 10% fetal bovine serum and 1% penicillin–streptomycin in an atmosphere of 5% CO_2_ at 37°C.

L-O2 cells were seeded in 96-well plates at 5 × 10^4^ cells per well to determine the effect of hesperetin on L-O2 cells. The cells were treated with hesperetin (25, 50, 100, 200, or 400 μmol/L) for 24 h. Then, 20 μL of 3-(4, 5-dimethylthiazol-2-yl)-2,5-diphenyltetrazolium bromide (MTT) was added to the 96-well plates, and incubation for 4 h in the dark allowed. Dimethyl sulfoxide (200 μL) was added to dissolve the remaining formazan crystals of MTT, and agitation for 15 min carried out. Cell viability was measured at 492 nm using a multimode microplate reader (Flexstation^™^ 3; Molecular Devices).

The same method was used to determine the effect of hesperetin on HEK293T cells. Cells were seeded in 96-well plates at 1.5 × 10^4^ cells per well and treated with hesperetin (25, 50, or 100 μmol/L) for 24 h. Then, cell viability was measured as described above.

### 2.10 Measurement of uric acid levels in L-O2 cells

L-O2 cells were incubated with hesperetin (i.e., 25, 50, or 100 μmol/L) or allopurinol (100 μmol/L) for 24 h. Then, cells were washed gently with phosphate-buffered saline (PBS). After incubated with hypoxanthine (800 μmol/L) for 24 h, xanthine oxidase (4 μL/well) was added at a final concentration of 0.3 U/g, and incubation for 1 h in the dark permitted, followed by extraction of the supernatant or protein. Experiments were repeated at least thrice. The uric acid content of cell supernatants was measured using the uric acid kit.

### 2.11 Determination of uric acid levels in HEK293T cells

As reported by W. D. Chen and Y. L. Zhao (Chen et al., 2020), HEK293T cells were resuspended with DMEM and adjusted to 1.5 × 10^4^ cells per well seeded in 96-well plates. Then, the cell supernatants of each well were discarded and hesperetin (6.25, 12.5, 25, or 50 μmol/L) added to the hesperetin group after inoculation for 24 h. The supernatant of each well was discarded 24 h after dosing. Sodium urate (SU) (8 mg/dL) was added to cells. The uric acid content of the supernatants and broken cells was detected after incubation for 24 h in a 37°C incubator. Experiments were repeated at least thrice. The uric acid content of cell supernatants was measured using the uric acid kit.

### 2.12 Western blotting

After pretreatment of cells, they were rinsed with pre-cooled PBS, and lysed in high-efficiency radioimmunoprecipitation (RIPA) tissue/cell lysate (lysate: phenylmethylsulfonyl fluoride (PMSF) = 100:1) on ice for 20 min. After scraping, the cell lysate was centrifuged at 15,000 g for 10 min at 4°C with the supernatant collected. Protein from liver tissues and kidney tissues was extracted using RIPA tissue/cell lysate, homogenized for 2 min, and centrifuged at 1,036 *g* for 10 min at 4°C. The supernatant fractions from cells and tissues were collected using 1.5-mL centrifuge tubes. The supernatant with equal amount of protein was mixed with SDS-PAGE loading buffer and immediately heated at 100°C for 10 min. Protein samples were separated by sodium dodecyl sulfate-polyacrylamide gel electrophoresis using 8%–12% polyacrylamide gels, and transferred to polyvinylidene fluoride (PVDF) membrane. After blocked non-binding sites with 5% skimmed milk for 1 h, the membrane was incubated overnight with specific antibodies in PBS: XOD (1:1,000), NLRP3 (1:1,000), IL-18 (1:200), TLR4 (1:200), NF-κB p65 (1:1,000), OAT1 (1:1,000), OAT3 (1:1,000), OCT1 (1:1,000), OCT2 (1:1,000), FOXO3a (1:1,000), MnSOD (1:1,000) or *β*-tubulin (1:1,000). Then, the blots washed thrice with tris-buffered saline with 0.1% Tween-20 (TBST) for 5-min each, and incubated with HRP-conjugated goat anti-rabbit IgG (1:5,000) or anti-mouse IgG (1:5,000) diluted with TBST for 1 h at room temperature. After three 5-min washes with TBST, images were acquired using a Pro-light HRP Chemiluminescence Kit (Tiangen Biotech, Beijing, China) on a FluorChem E System (Protein Simple, Santa Clara, CA, United States).

### 2.13 Statistical analyses

Statistical analyses were undertaken with Prism 5 (GraphPad, La Jolla, CA, United States), using mean ± standard error of the mean. Dunnett’s tests were used for post-hoc evaluations and *p* < 0.05 was considered statistically significant.

## 3 Results

### 3.1 Hesperetin exhibited potent xanthine oxidase inhibition *in vitro*

Allopurinol inhibited xanthine oxidase activity in a dose-dependent manner with an IC_50_ of 6.044 μmol/L, whereas the IC_50_ of the O^2-^ level was 11.2 μmol/L (Figures 1B,C). Hesperetin inhibited xanthine oxidase activity with an IC_50_ of 110.4 μmol/L, whereas the IC_50_ of the O^2-^ level was 317.6 μmol/L (Figures 1D,E). The inhibitory activity on xanthine oxidase elicited by hesperetin is consistent with the literature, though the IC_50_ of hesperetin was higher than that of allopurinol (Haidari et al., 2009). Hesperetin appeared to be a potent inhibitor of xanthine oxidase activity.

### 3.2 Hesperetin reduced the serum parameters and hepatic xanthine oxidase activity in yeast extract-induced hyperuricemia in mice

**FIGURE 3:**
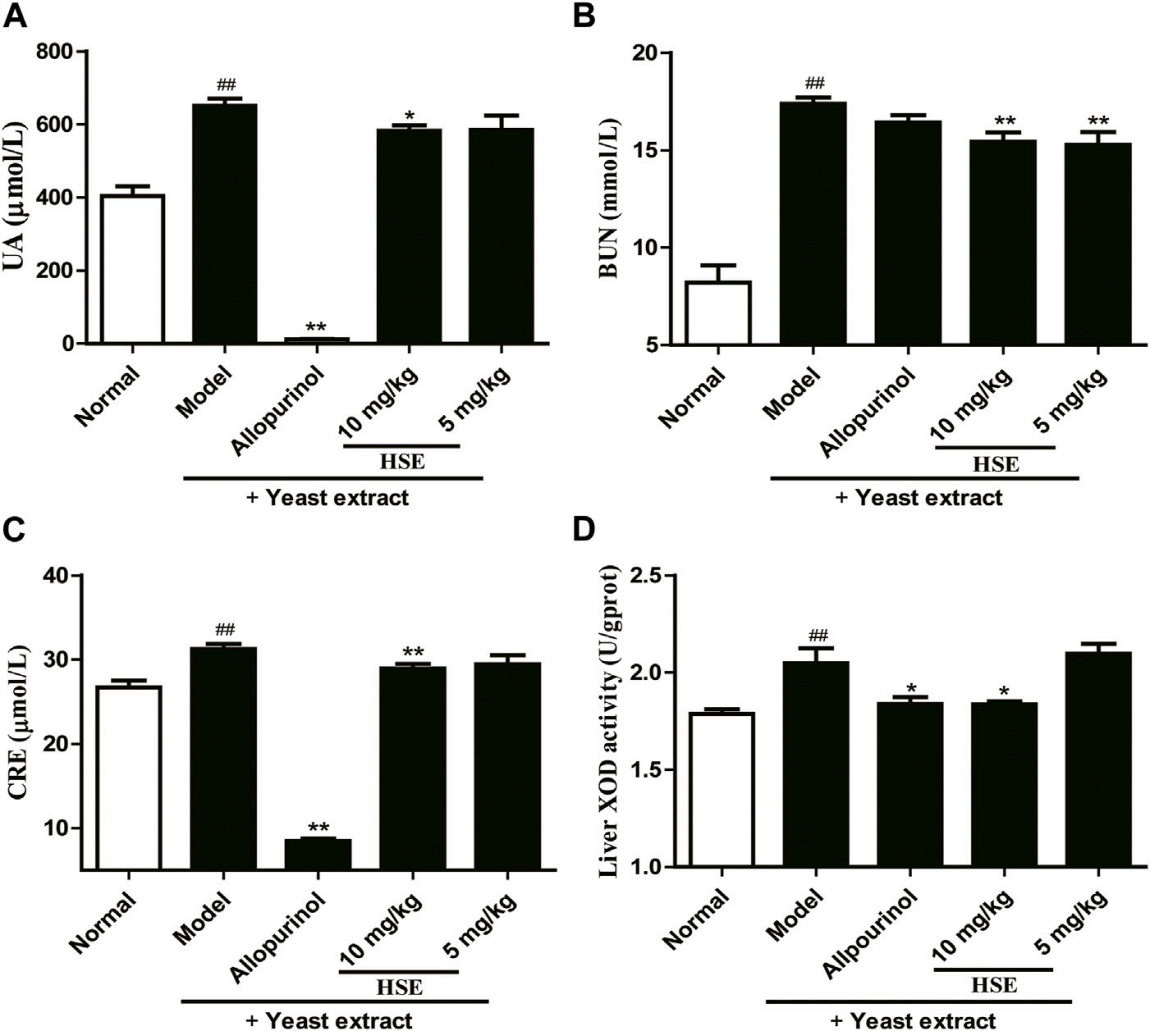
Effects of hesperetin (HSE) on serum parameters and hepatic xanthine oxidase (XOD) activity in mice with hyperuricemia induced by yeast extract. **(A)** Uric acid (UA) **(B)** Blood urea nitrogen (BUN) **(C)** Serum creatinine (CRE) **(D)** Hepatic xanthine oxidase (XOD) activity. The dose of allopurinol was 10 mg/kg *in vivo* experiments. Data are the mean ± SEM (n = 10). ^##^ *p* < 0.01 *versus* the normal group; *p < 0.05 and **p < 0.01 *versus* the model group. HSE, hesperetin; UA, uric acid; BUN, blood urea nitrogen; CRE, serum creatinine; XOD, xanthine oxidase.

Compared with normal mice, mice in which hyperuricemia had been induced using yeast extract showed markedly increased serum levels of uric acid, urea nitrogen, and creatinine and hepatic xanthine oxidase activity, indicating mild damage to kidney function (Figure 3). Serum levels of uric acid, urea nitrogen, and creatinine and hepatic xanthine oxidase activity were reduced markedly after hesperetin treatment (10 and 5 mg/kg) (*p* < 0.05/0.01), and the effect was similar to that of allopurinol. These results suggested that hesperetin not only reduced the serum uric acid level and hepatic xanthine oxidase activity but also protected the kidneys from damage caused by high-purine diets.

### 3.3 Hesperetin suppressed expression of xanthine oxidase protein and downregulated activation of the NLRP3 inflammasome in mice suffering from yeast extract-induced hyperuricemia

**FIGURE 4:**
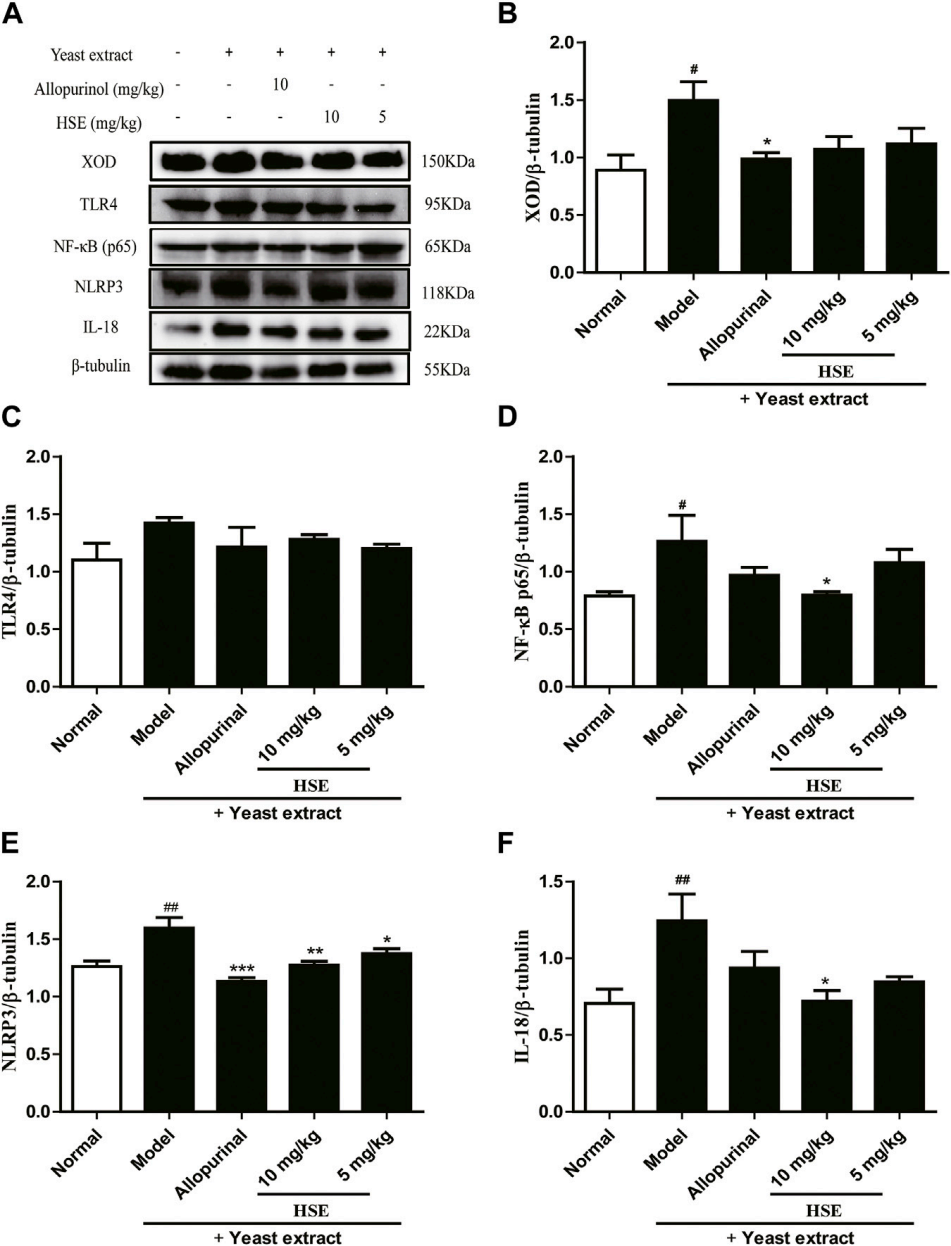
Effects of hesperetin (HSE) on protein levels in mice liver with hyperuricemia induced by yeast extract. Protein expression of XOD **(B)**, TLR4 **(C)**, NF-κB p65 **(D)**, NLRP3 **(E)**, IL-18 **(F)** and *β*-tubulin were determined by Western blotting quantified using ImageJ. The dose of allopurinol was 10 mg/kg *in vivo* experiments. Data are the mean ± SEM (n = 10). ^#^p < 0.05 and ^##^ *p* < 0.01 *versus* the normal group; *p < 0.05, **p < 0.01 and ***p < 0.001 *versus* the model group. HSE, hesperetin; XOD, xanthine oxidase; TLR 4: Toll-like receptor 4; NF-κB p65: nuclear factor-κB p65; NLRP3: nucleotide binding oligomerization domain-like receptor family pyrin domain-containing 3; IL-18: interleukin-18.

Expression of XOD (Figures 4A, B) and proteins associated with NLRP3 inflammasome signaling, such as TLR4 (Figure 4C), NF-κB (Figure 4D), NLRP3 (Figure 4E) and IL-18 (Figure 4F), was increased in the liver tissues of mice suffering from hyperuricemia induced by yeast extract compared with those in the normal group. Expression of all of these moieties was downregulated after hesperetin treatment for 14 successive days compared with that in the model group (*p* < 0.05). Similar results were observed in the allopurinol group, of them, the downregulation of IL-18 inflammatory signaling protein was inferior to that of hesperetin.

### 3.4 Effect of hesperetin on oxidative stress parameters and proteins in liver

**FIGURE 5:**
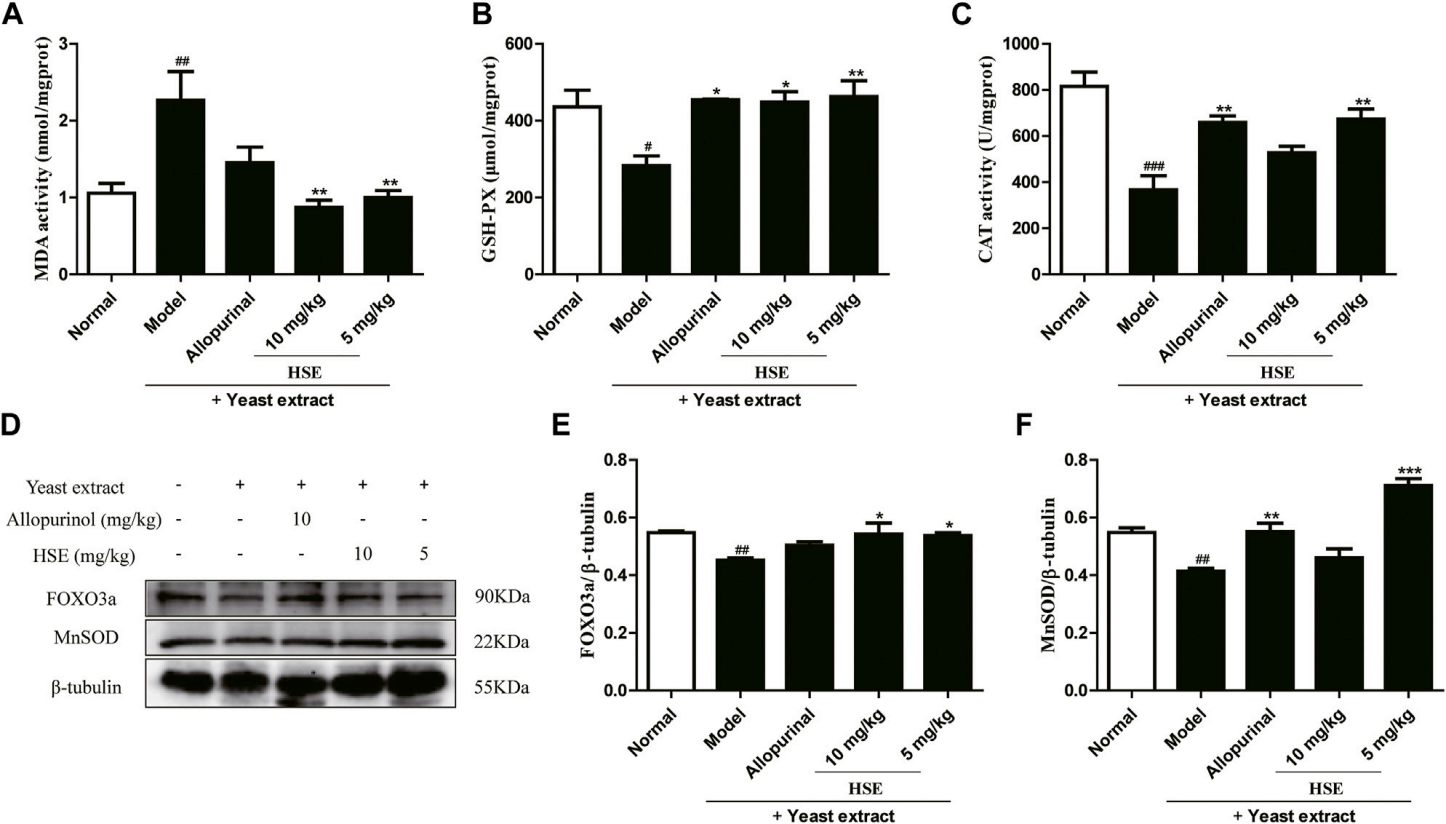
Effects of hesperetin (HSE) on oxidative stress in mice liver with hyperuricemia induced by yeast extract. **(A)** MDA activity **(B)** GSH-PX **(C)** CAT activity. Protein expression of FOXO3a **(E)**, MnSOD **(F)**, and *β*-tubulin were determined *via* Western blotting quantified with ImageJ. The dose of allopurinol was 10 mg/kg *in vivo* experiments. These data were from three separate experiments and expressed as the mean ± SEM. ^#^ *p* < 0.05, ^##^ *p* < 0.01 and ^###^ *p* < 0.001 *versus* the normal group; *p < 0.05, **p < 0.01 and ***p < 0.001 *versus* the model group. HSE, hesperetin; MDA, malondialdehyde; CAT, catalase; GSH-PX, glutathione peroxidase; FOXO3a, forkhead box O3a; MnSOD, manganese superoxide dismutase.

The model group showed elevated MDA in mice liver compared to the normal group, which was significantly reduced (*p* < 0.01) (Figure 5A) by HSE administration. Next, we further analyzed the endogenous antioxidant levels in liver. Compare the normal group, the model group remarkably diminished the levels of GSH-PX and CAT activities. Interestingly, 10 mg/kg of HSE obviously increased the GSH-PX (*p* < 0.01) (Figure 5B) and CAT activities (*p* < 0.01) (Figure 5C) compared to the model group. As expected, this data suggests that HSE treatment significantly reversed the oxidative stress parameters in the liver and prevented the development of its conditions. In addition, compared with the model group, HSE could significantly upregulate the protein levels of FOXO3a and MnSOD, which further indicated that HSE could improve the level of oxidative stress caused by yeast extract.

### 3.5 Hesperetin improved changes in liver histopathology

**FIGURE 6:**
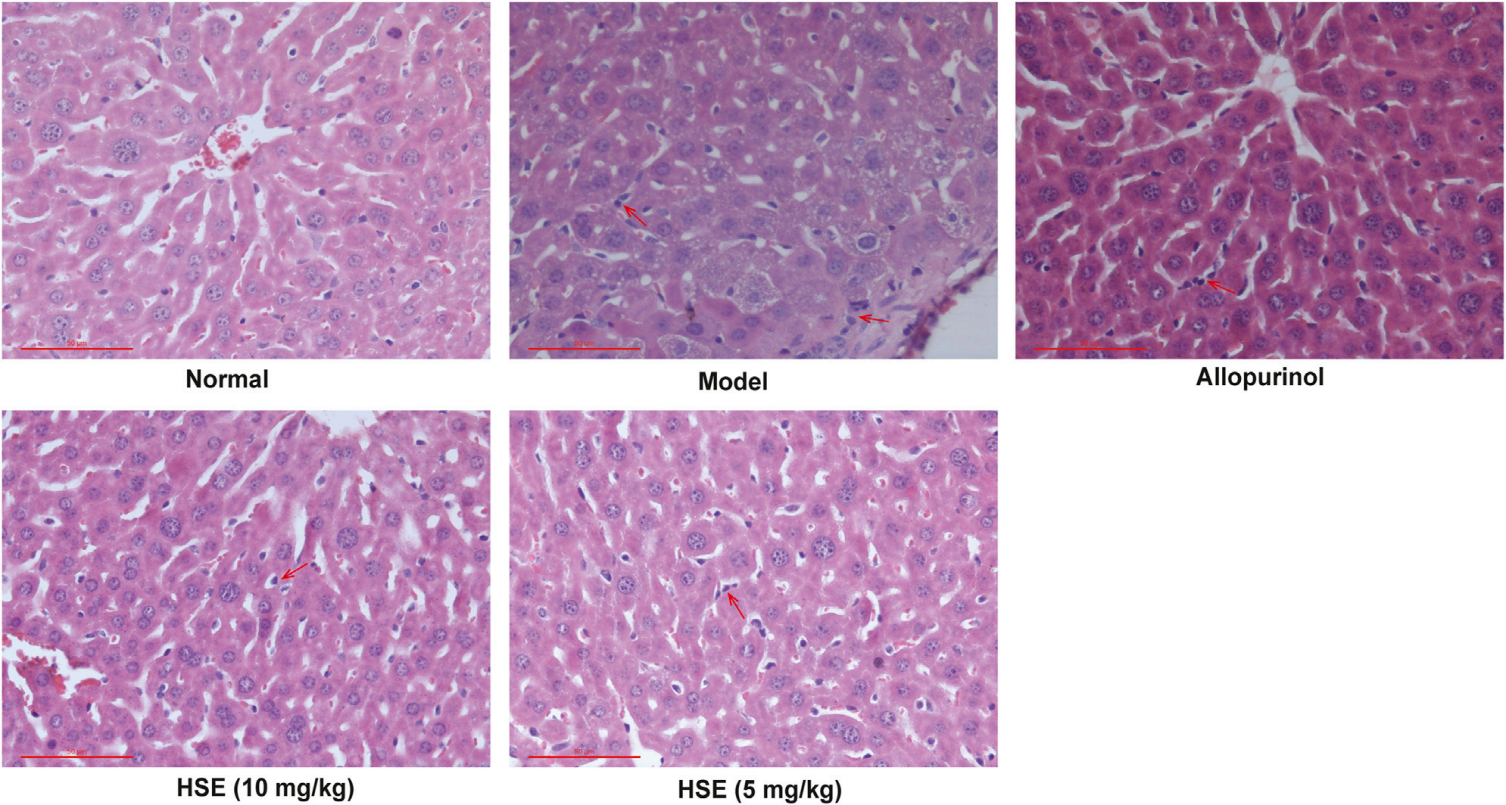
Hepatoprotective effects of hesperetin (HSE) on liver morphology in mice with hyperuricemia induced by yeast extract. Liver sections subjected to hematoxylin and eosin staining (n = 10) are presented at a magnification of ×400. Scale bar, 50 μm. Hesperetin (HSE) (5 and 10 mg/kg) and allopurinol (10 mg/kg) were administered, respectively, to mice in addition to yeast extract treatment. HSE, hesperetin. Inflammatory cell infiltration was point out with red arrows.

Hematoxylin and eosin staining of liver sections (Figure 6) from the yeast extract-induced hyperuricemia group showed obvious pathologic changes: unclear structure of hepatic lobules, degeneration of hepatocyte vacuoles, and inflammatory-cell infiltration. These pathologic changes could be alleviated by treatment with hesperetin (10 and 5 mg/kg) (Figure 6). The positive allopurinol group showed similar improvements in inflammatory cell infiltration and hepatocyte vacuolation in liver tissue. These results demonstrated that hesperetin exerted a hepatoprotective effect in mice with yeast extract-induced hyperuricemia.

### 3.6 Hesperetin reduced the serum parameters and enhanced expression of uric acid-excreting transporters in mice with potassium oxonate-induced hyperuricemia

**FIGURE 7:**
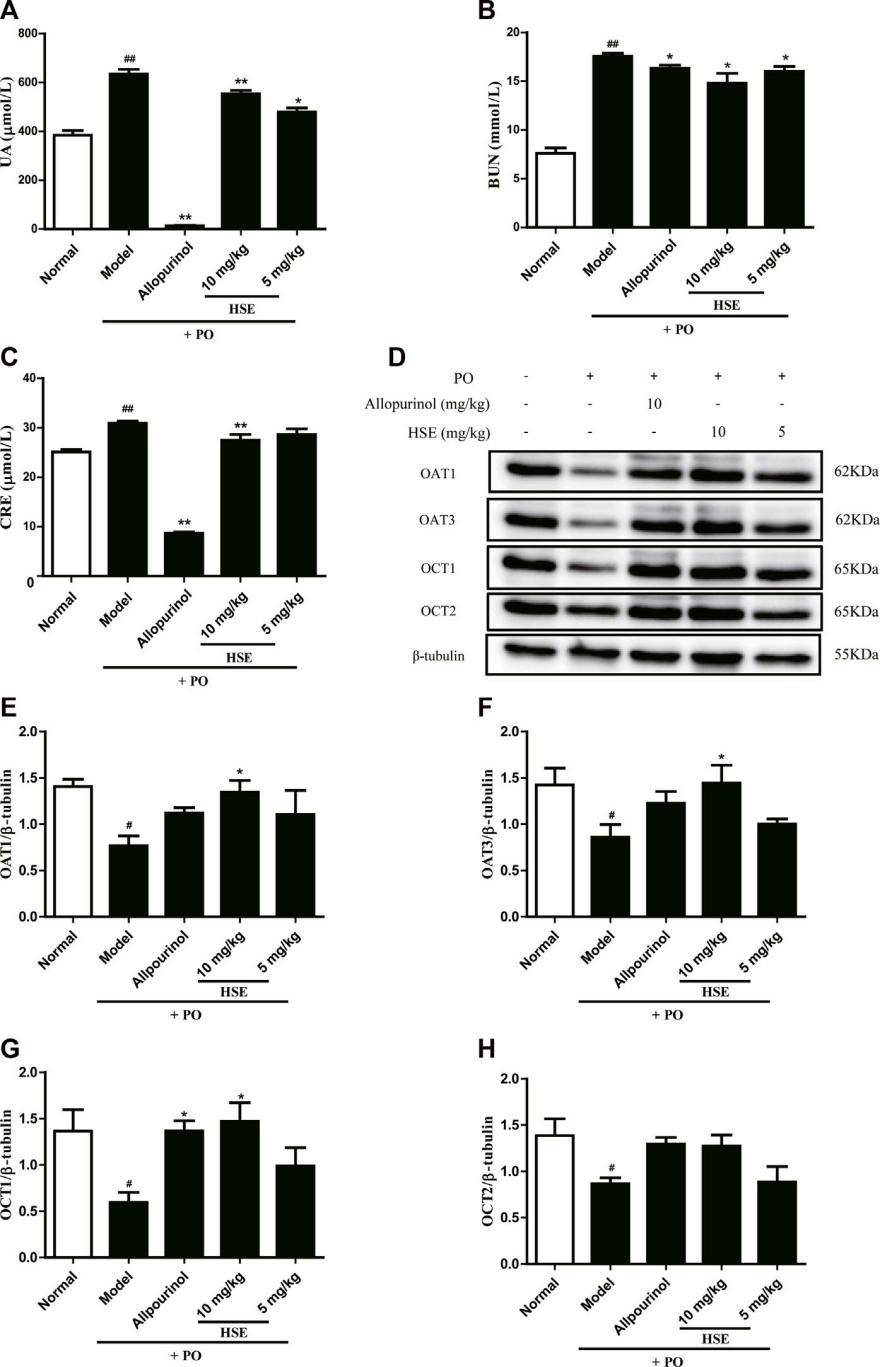
Effects of hesperetin (HSE) on serum parameters and protein levels in mice with hyperuricemia induced by potassium oxonate. **(A)** Uric acid (UA) **(B)** Blood urea nitrogen (BUN) **(C)** Serum creatinine (CRE). Protein expression of OAT1 **(E)**, OAT3 **(F)**, OCT1 **(G)**, OCT2 **(H)**, and *β*-tubulin were determined *via* Western blotting quantified with ImageJ. The dose of allopurinol was 10 mg/kg *in vivo* experiments. These data were from three separate experiments and expressed as the mean ± SEM. ^#^ *p* < 0.05 and ^##^ *p* < 0.01 *versus* the normal group; *p < 0.05 and **p < 0.01 *versus* the model group. HSE, hesperetin; UA, uric acid; BUN, blood urea nitrogen; CRE, serum creatinine; PO, potassium oxonate; XOD, xanthine oxidase; OAT1, organic anion transporter 1; OAT3, organic anion transporter 3; OCT1, organic cationic transporters 1; OCT2, organic cationic transporters 2.

Single injection with potassium oxonate significantly increased serum levels of uric acid in mice with hyperuricemia compared with that in normal mice (Figure 7), a result which is consistent with data from Tung Y. T (Tung et al., 2015). Hesperetin (10 and 5 mg/kg) could reduce serum uric acid levels and improve renal injury in mice with hyperuricemia 7 days after intraperitoneal injection (Figures 7A–C). Moreover, hesperetin (10 mg/kg) could upregulate expression of OAT1 (Figure 7E), OAT3 (Figure 7F), OCT1 (Figure 7G), and OCT2 (Figure 7H) proteins. Interestingly, allopurinol reduced serum levels of uric acid and improved renal injury, but weakly regulated expression of uric acid-excretion proteins compared with that of hesperetin at 10 mg/kg. These results indicated that hesperetin could reduce serum uric acid levels markedly through upregulation of expression of OAT1/OAT3/OCT1/OCT2 proteins to promote excretion of uric acid.

### 3.7 Hesperetin reduced uric acid levels in L-O2 cells

**FIGURE 8:**
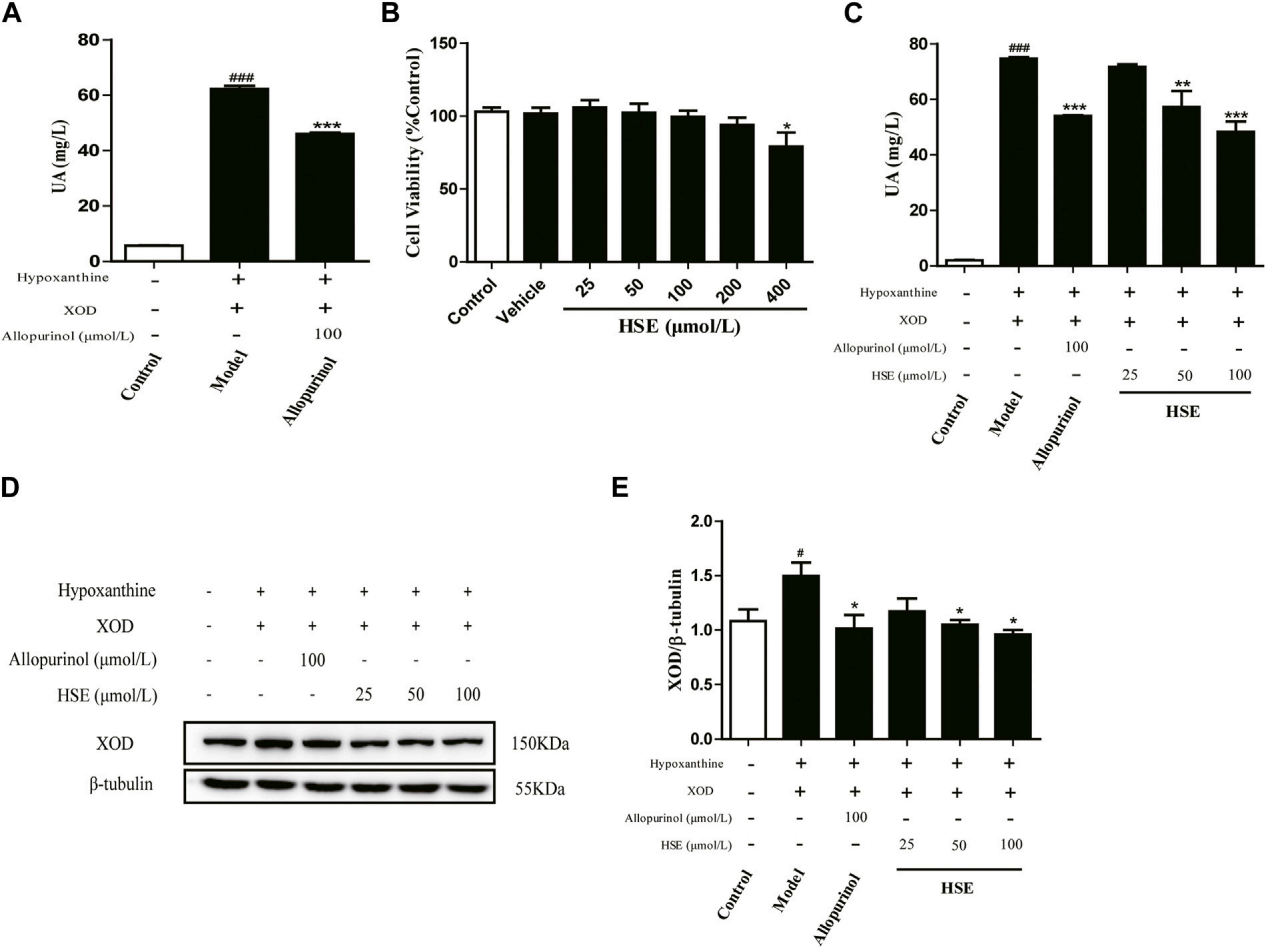
Hesperetin (HSE) inhibited the production of uric acid (UA) *in vitro*. Establishment of a high uric acid (UA) model in cells **(A)** and effects of hesperetin (HSE) on the viability of L-O2 cells **(B)**; Uric acid (UA) level **(C)**; the band of XOD protein **(D)** and quantitative analysis **(E)**. L-O2 cells were seeded in six-well plates overnight, incubated with 2 mL of hesperetin (HSE) (25, 50, or 100 μmol/L) or allopurinol (100 μmol/L) for 24 h, followed by incubation with hypoxanthine (800 μmol/L) for 24 h and addition of xanthine oxidase (4 μL/well). Data are the mean ± SEM (n = 3) and representative of three independent experiments. ^#^ *p* < 0.05 and ^###^ *p* < 0.001 *versus* the control group; *p < 0.05, **p < 0.01 and ***p < 0.001 *versus* the model group. HSE, hesperetin; UA, uric acid; XOD, xanthine oxidase.

Uric acid is produced in the liver. A model of high uric acid in L-O2 hepatocytes was induced by a combination of hypoxanthine and xanthine oxidase. The level of uric acid in the model group was obviously more than that in the control group, so the hyperuricemia model had been established (Figure 8A). Then, the MTT assay was used to evaluate the action of hesperetin against hyperuricemia in L-O2 cells (Figure 8B). Interestingly, hesperetin exerted no obvious inhibition at 200 μmol/L (Figure 8B), but the uric acid level was decreased by 34% at 100 μmol/L of hesperetin (*p* < 0.001) (Figure 8C). Furthermore, hesperetin reduced expression of XOD protein compared with that in the model group (*p* < 0.05) (Figures 8D, E). These results indicated that hesperetin could reduce uric acid levels by inhibiting expression of XOD protein in a hyperuricemia model.

### 3.8 Hesperetin promoted the transport of uric acid from inside to outside of HEK293T cells induced by sodium urate

**FIGURE 9:**
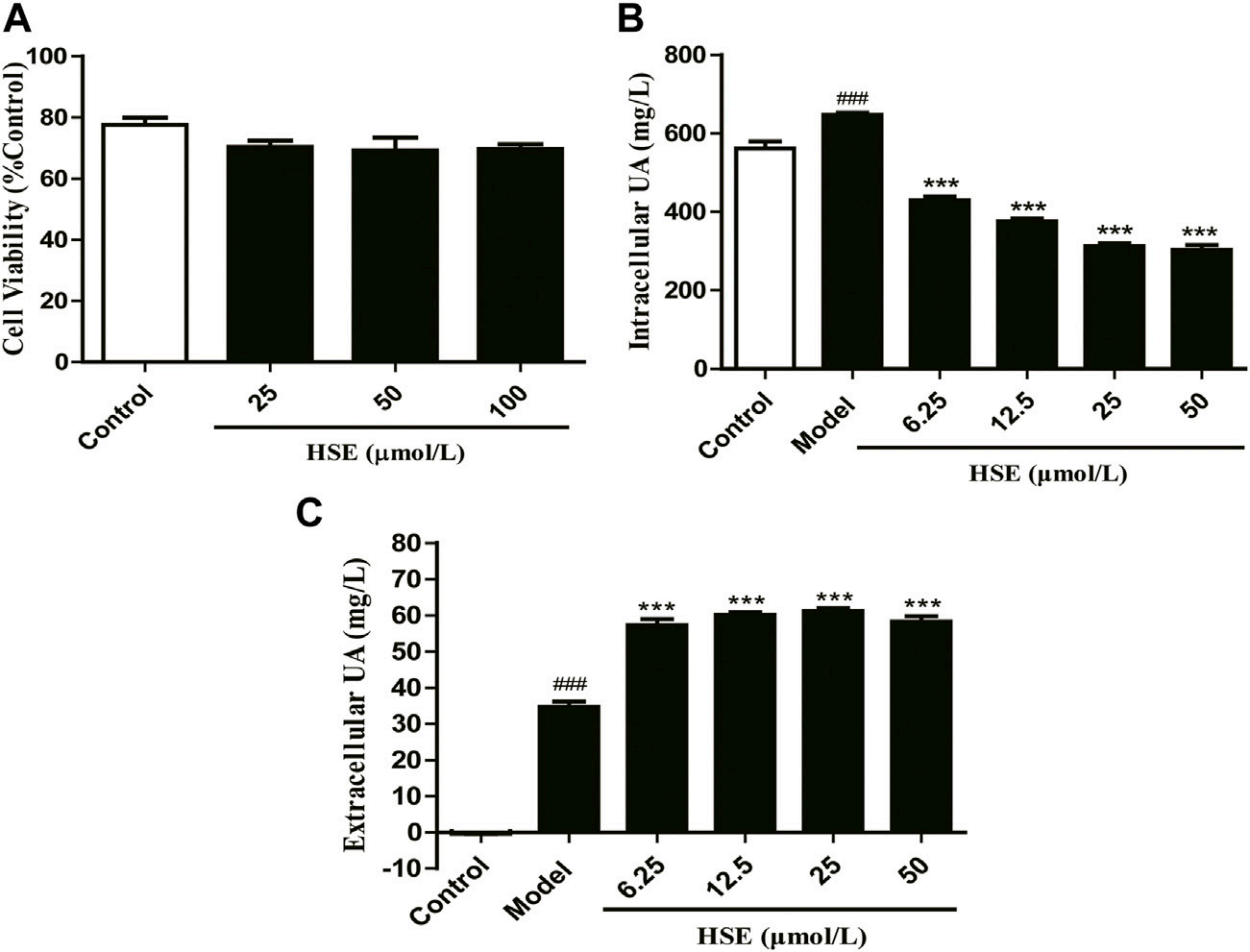
Hesperetin (HSE) promoted uric acid (UA) excretion *in vitro*. Effects of hesperetin (HSE) on the viability of HEK293T cells **(A)** and intracellular uric acid (UA) level **(B)**, and extracellular uric acid (UA) level **(C)**. Data are the mean ± SEM (n = 3) and representative of three independent experiments. ^###^ *p* < 0.001 *versus* the control group; ***p < 0.001 *versus* the model group. HSE, hesperetin; UA, uric acid.

We wished to assess the effect of hesperetin on uric acid excretion. Hence, we used a sodium urate-induced model of hyperuricemia in HEK293T cells to detect changes in intracellular and extracellular uric acid levels. Hesperetin (25, 50, or 100 μmol/L) had hardly effect on the survival of HEK293T cells (*p* > 0.05) (Figure 9A). Compared with the control group, the intracellular uric acid content of the model group was remarkably increased (*p* < 0.001) (Figure 9B), showing that the model could be used in experimental studies. The intracellular uric acid level decreased gradually as the hesperetin concentration increased (*p* < 0.001) (Figure 9B), whereas the extracellular uric acid level increased gradually (*p* < 0.001) (Figure 9C) compared with that in the model group. These results showed that hesperetin could promote uric acid excretion from inside to outside of HEK293T cells.

## 4 Discussion

Many edible plants have medicinal values for promoting human health, and hesperetin is an important bioactive agent from edible plants (Luo et al., 2020). This study is the first to explore the mechanism of action of hesperetin on uric acid metabolism. Xanthine oxidase catalyzes xanthine to produce uric acid as well as superoxide anions (Lu et al., 2013). Excessive production of superoxide anions in the body can cause oxidative stress, which increases the risk of complications of hyperuricemia (Enroth et al., 2000; Ives et al., 2015). Therefore, antioxidant or anti-inflammatory activities of compounds may have value in delaying the development of these diseases. Hyperuricemia due to increased synthesis of uric acid is caused by increased xanthine oxidase activity, mainly due to excessive intake of purine-rich foods or uric acid precursors.

Mouse models of hyperuricemia have been widely used to provide valuable insights into uric-acid-lowering drugs. These models are highly diverse and can be duplicated by diet or pharmaceutical induction. Dietary Modification is widely used to induce elevated serum urate concentrations in mice. By increasing the precursors of uric acid synthesis, such as yeast, which are rich in proteins, nucleic acids, *etc.*, can be completely hydrolyzed into organic bases (including purines and pyrimidines) and phosphoric acid *in vivo*. Therefore, when there are more yeasts in the body, the normal purine metabolism is disturbed by the increase of xanthine oxidase activity and the acceleration of uric acid production, resulting in a large amount of uric acid (Chen et al., 2006). This model resembles human hyperuricemia induced by a high-protein diet. Therefore, a hyperuricemia mice model using yeast extract was created, and the effect of hesperetin on uric acid synthesis studied.

We showed that serum levels of uric acid, creatinine, urea nitrogen, and hepatic xanthine oxidase activity were decreased significantly and expression of XOD protein downregulated after hesperetin treatment. Studies have shown that increased serum levels of uric acid are accompanied by activation of inflammasome even in the absence of gout (Ghaemi-Oskouie and Shi, 2011; Grainger et al., 2013). The increase of uric acid is accompanied by the generation of oxidative stress, and meanwhile, the damaged inflammatory cells release more reactive oxygen species, which further leads to the increase of oxidative stress level (Liu et al., 2021). MDA is regarded as a biomarker of oxidative damage, and its excessive production will accelerate oxidative stress. GSH as a cellular antioxidant marker; CAT removes hydrogen peroxide from the body and is one of the key enzymes in biological defense systems (Li et al., 2019; Wang et al., 2022). FOXO3a can regulate the expression of related proteins in the cellular defense mechanism against oxidative stress, promote the scavenging of reactive oxygen species and malondialdehyde (MDA), and increase the activity of the antioxidant enzyme MnSOD to reduce oxidative stress (Zhang et al., 2022; Kang et al., 2023). Interestingly, hesperetin exerts a hepatoprotective effect in our hyperuric acid model by reducing oxidative stress (CAT, GSH, MDA parameters, FOXO3a-MnSOD signaling pathway) and down-regulating the TLR4-NLRP3 inflammatory pathway, data which are consistent with findings from the study (Wan et al., 2020). Next, a hyperuricemia cell model was created by hypoxanthine and xanthine oxidase to explore the effect of hesperetin on uric acid synthesis. We showed that the uric acid level and protein expression of XOD were downregulated after hesperetin treatment, which is consistent with the role of hyperuricemia induced by yeast extract in animals. These data indicated that HSE inhibited XOD activity *in vitro* and *in vivo*, slow down its catalytic rate of hypoxanthine to uric acid, and reduce the production of uric acid and reactive oxygen species.

Uricase is an enzyme that converts uric acid to allantoin which is much more soluble than uric acid. Given that the gene encoding uricase is inactivated in humans but not in mice. Administration of a uricase inhibitor——oxonic acid or potassium oxonate, a selectively competitive uricase inhibitor, blocks the effect of uricase, is a commonly used method to induce mild hyperuricemia in mice. Different from yeast extract-induced hyperuricemia model, its target tissue is kidney, and the research focus is uric acid transporter and uric acid-related metabolic diseases (Feng et al., 2022). Thus, a hyperuricemia model was created using a single injection of potassium oxonate to study the effect of hesperetin on uric acid excretion. It has been reported that an increase in uric acid level is due to dysfunction of uric acid transporters, which are responsible for the reabsorption or excretion of uric acid (Xu et al., 2017). In recent years, the OAT1, URAT1, ABCG2, and GLUT9 have become potential targets for hyperuricemia treatment (Xu et al., 2017; Wu et al., 2020). In present research, hesperetin could significantly reduce uric acid levels by upregulating protein expression of OAT1, OAT3, OCT1, and OCT2. Uric acid is excreted mainly in the kidneys. Suboptimal excretion of uric acid would increase the burden of kidneys and renal injury. We measured the serum level of creatinine and urea nitrogen and the effect of hesperetin administration. However, histopathologic changes in the kidneys were not revealed using hematoxylin and eosin (data not shown). Subsequently, we measured the uric acid levels inside and outside of HEK293T cells induced by sodium urate. We found that the intracellular uric acid level decreased and extracellular uric acid level increased after hesperetin treatment, which further verified that hesperetin could promote uric acid excretion.

## 5 Conclusion

Hesperetin could significantly reduce the uric acid level and protect against hyperuricemia-associated liver damage. The mechanism of action of hesperetin was associated with inhibition of xanthine oxidase activity and protein expression, alteration of the MDA, GSH-PX and CAT content, downregulation of the TLR4-NLRP3 inflammasome signaling pathway, and upregulation of expression of FOXO3a, MnSOD, OAT1, OAT3, OCT1, and OCT2 proteins. This study validates the efficacy of hesperetin in hyperuricemia treatment and provides valuable insights into its potential biological mechanisms.

Based on the results of this study, we will investigate the effect of hesperetin on the complications of hyperuricemia (e.g., gout, nephritic syndrome) in future studies. Moreover, a natural product based on hesperetin used in lowering uric acid levels is worth developing.

## Data Availability

The original contributions presented in the study are included in the article/Supplementary Material, further inquiries can be directed to the corresponding authors.
