# Supplementary material for: Anti-hyperuricemia effect of hesperetin is mediated by inhibiting the activity of xanthine oxidase and promoting excretion of uric acid
Source: Front Pharmacol. 2023 Apr 12;14:1128699. doi: 10.3389/fphar.2023.1128699 (PMC10131109; doi:10.3389/fphar.2023.1128699)
Supplement: Supplementary file 1 [file Table2.DOCX]

**Western Blot original data for**

**Anti-hyperuricemia effect of hesperetin is mediated by inhibiting the activity of xanthine oxidase and promoting excretion of uric acid**

**Hyperuricemia model induced by yeast extract**

**IL-18-YE-(NLRP3)-1 and IL-18-YE-NLRP3-2 are the same blot.**

**NLRP3-YE-(GLUT9)-1 and NLRP3-YE-GLUT9-2 are the same blot.**

**XOD-YE-(ABCG2)-1 and XOD-YE-ABCG2-2 are the same blot.**


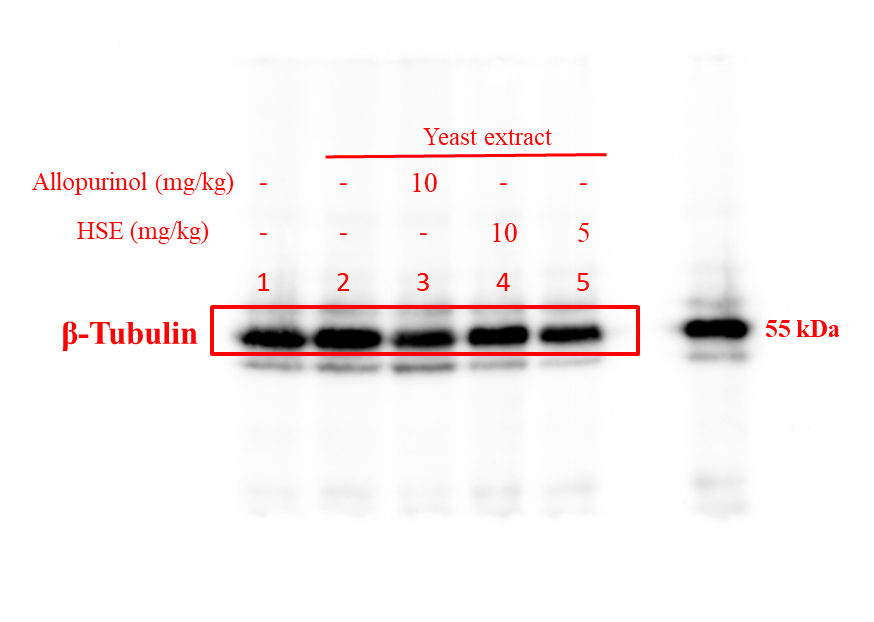


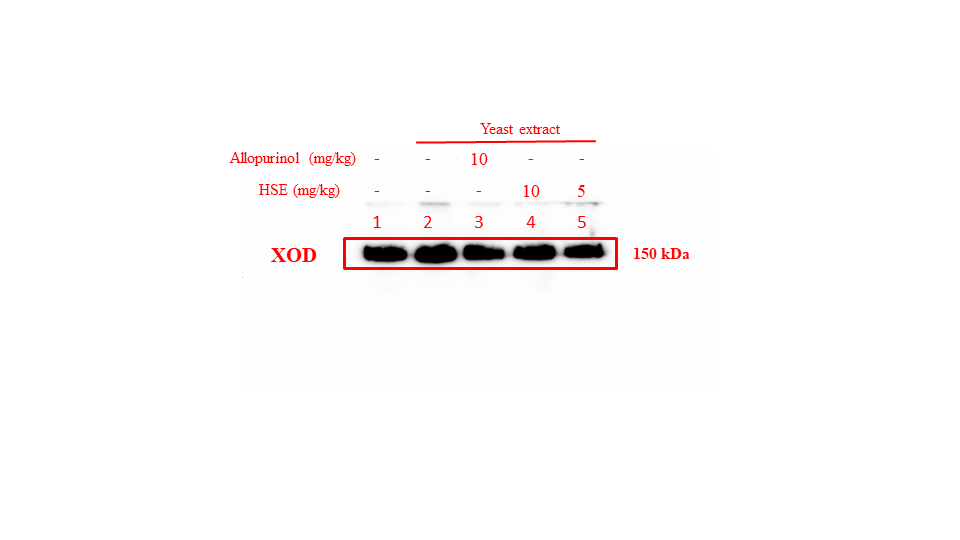


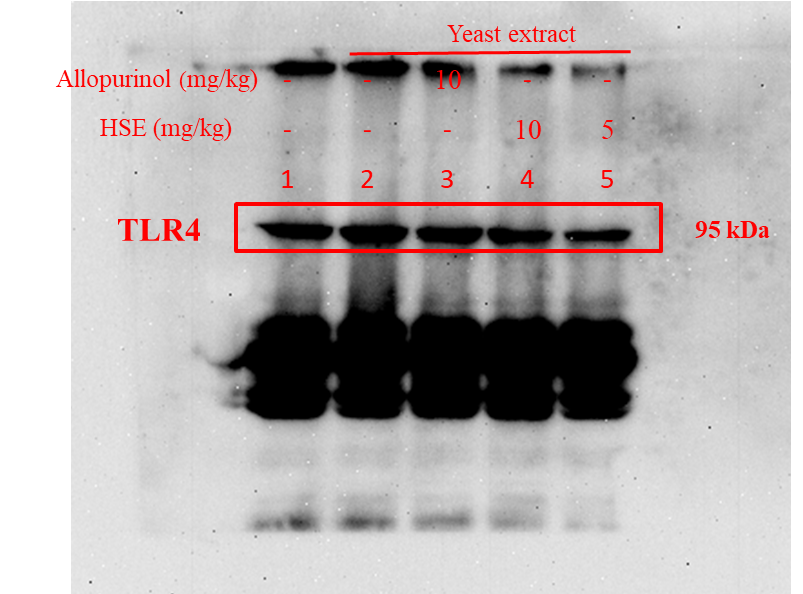


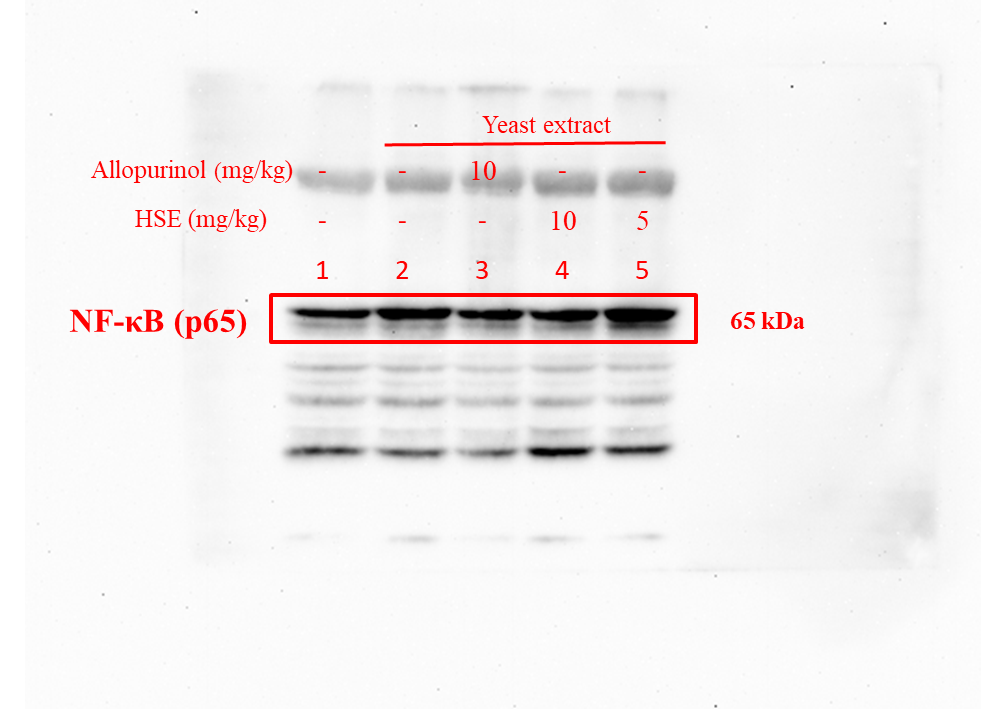


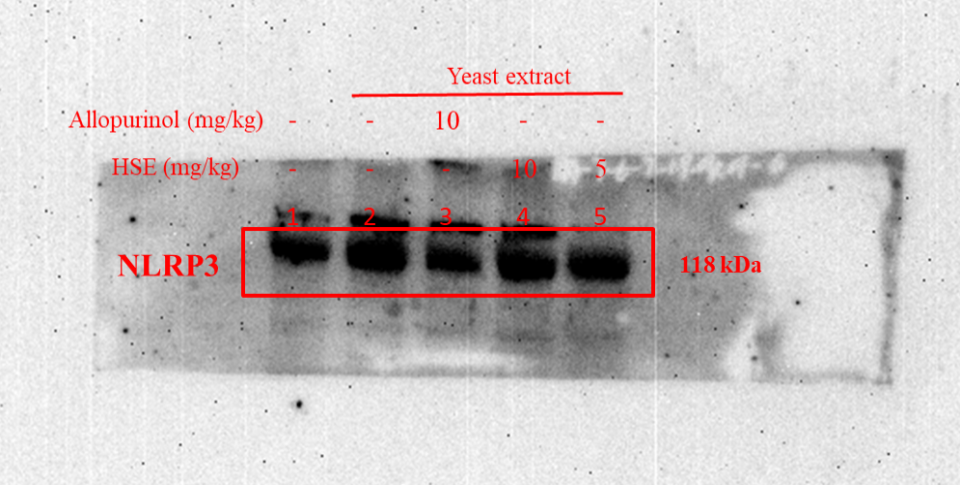


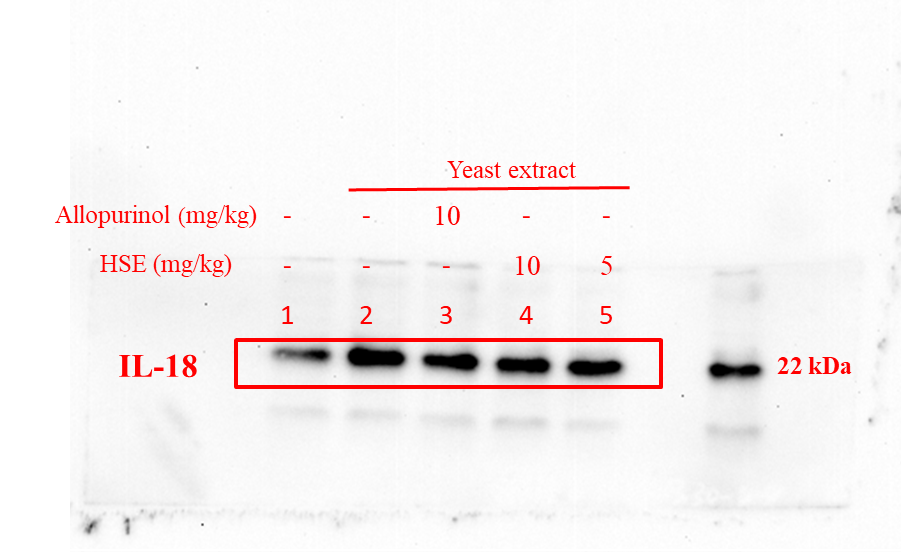


**Hyperuricemia model induced by potassium oxonate**
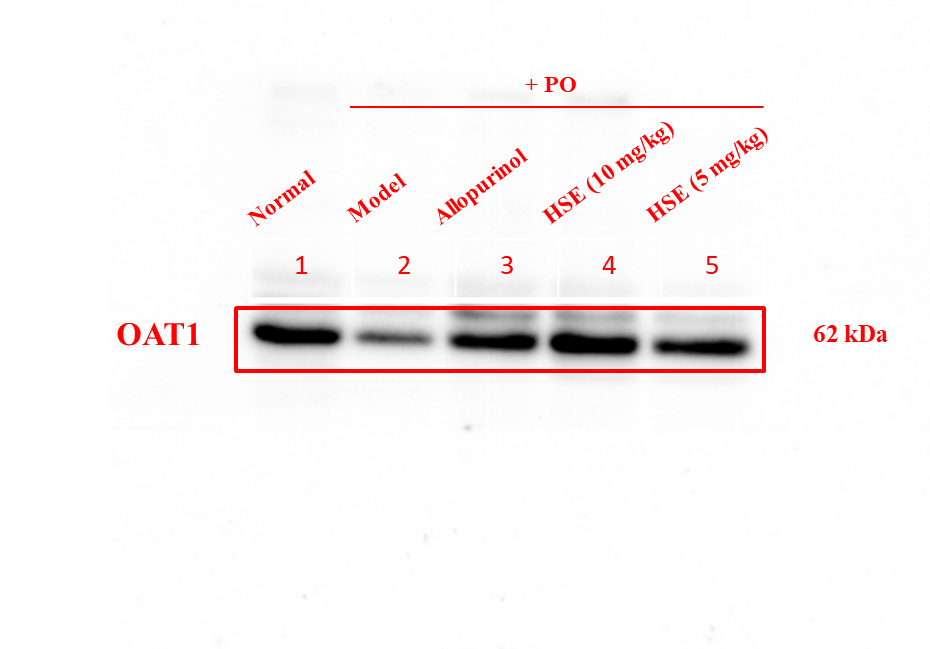

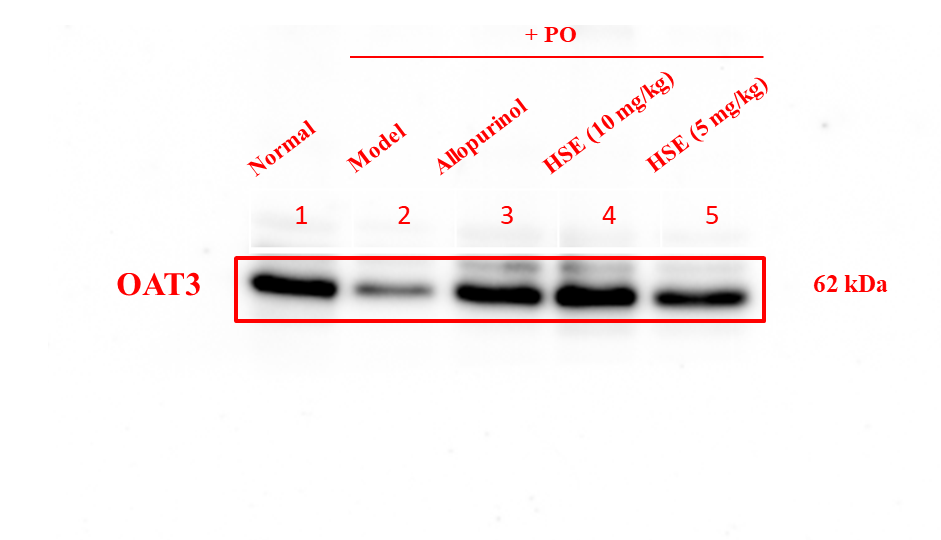

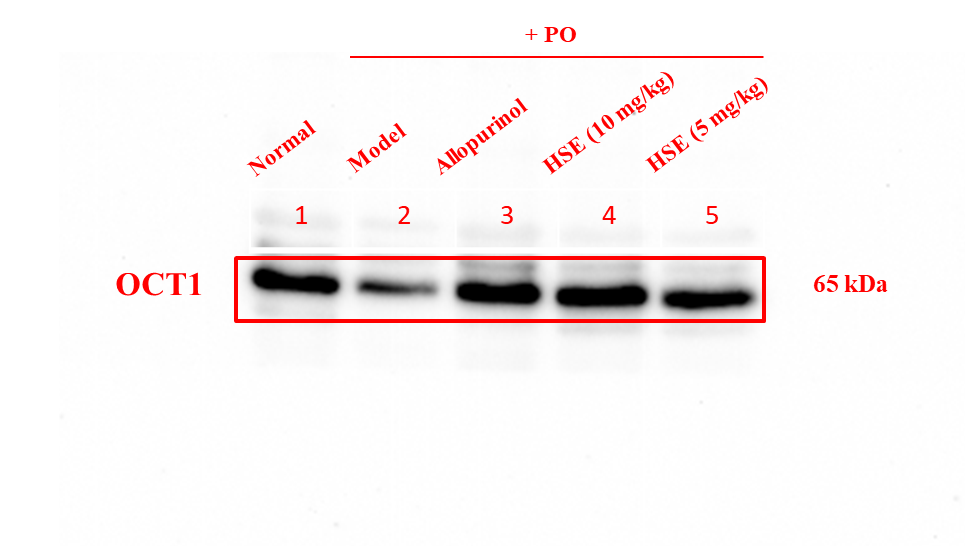


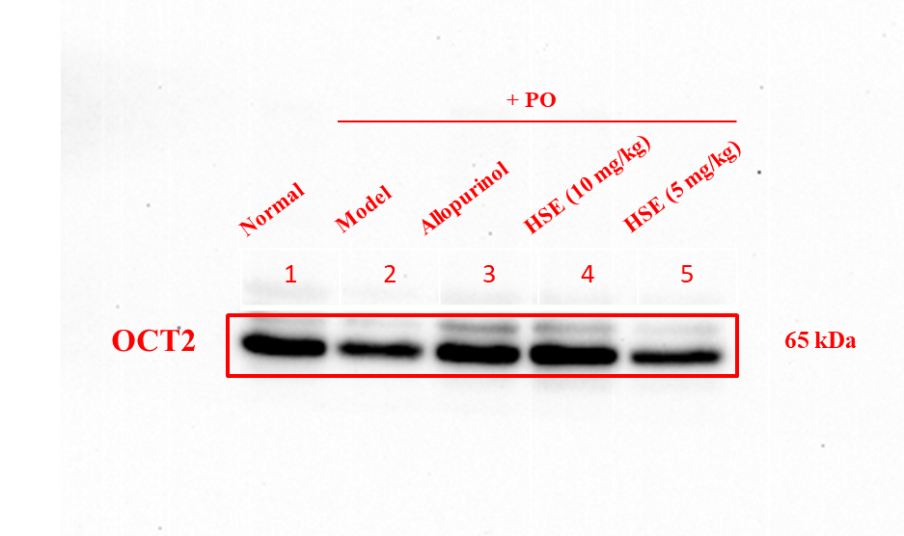


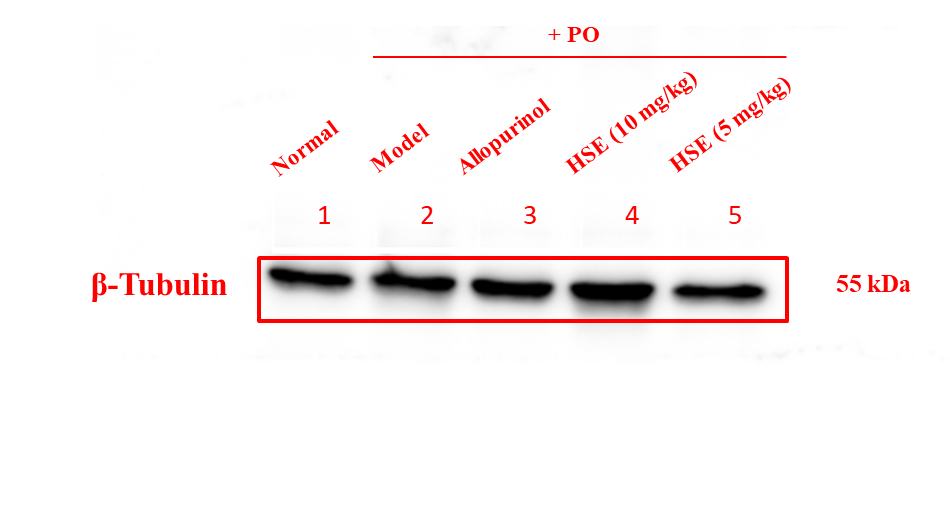


**Cells**

**
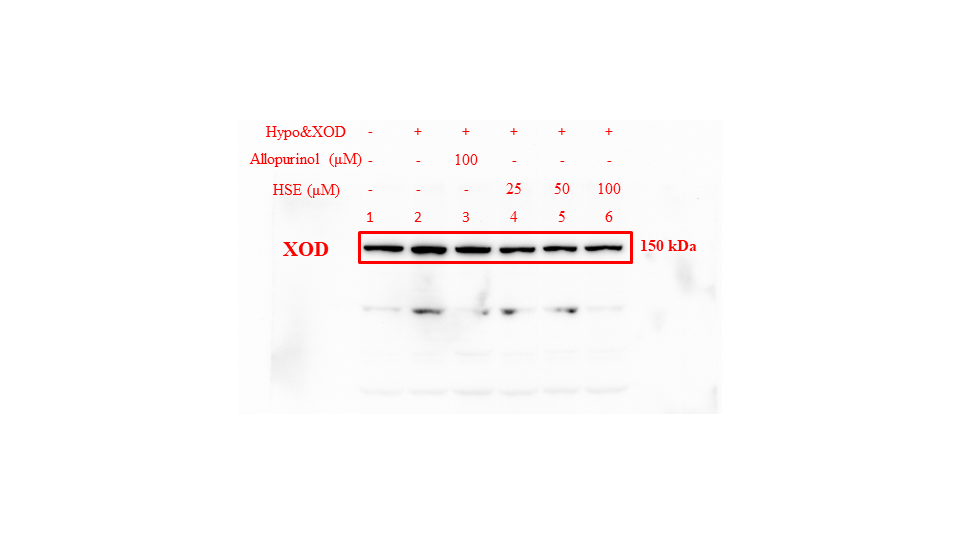

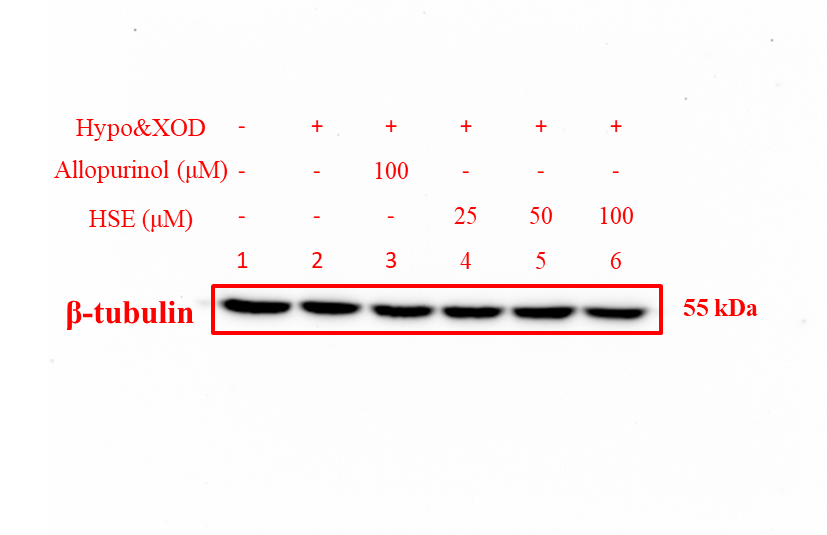
**

**Supplement**

**MnSOD-YE-(COX2)-1 and MnSOD-YE-COX-2 are the same blot.**

**FOXO3a-YE-(MnSOD)-1 and FOXO3a-YE-MnSOD-2 are the same blot.**

**
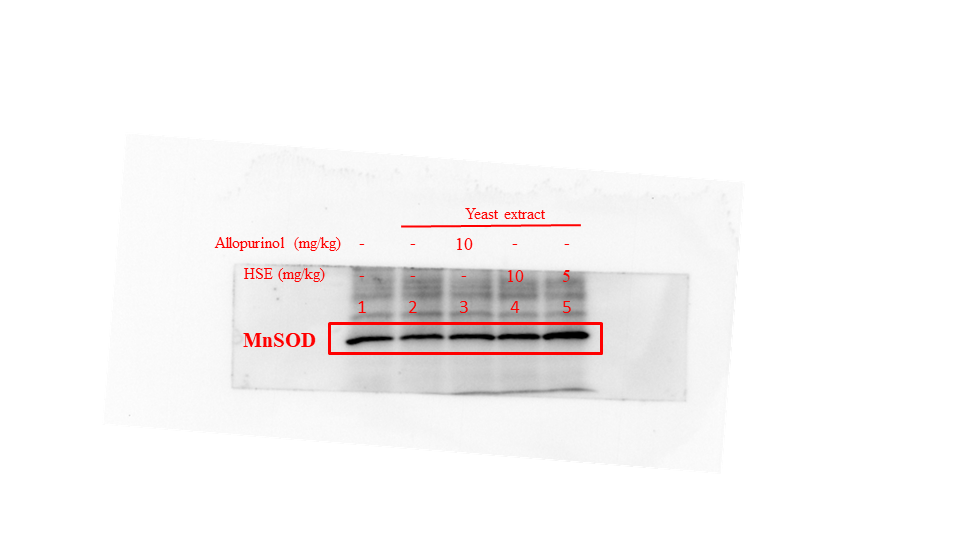

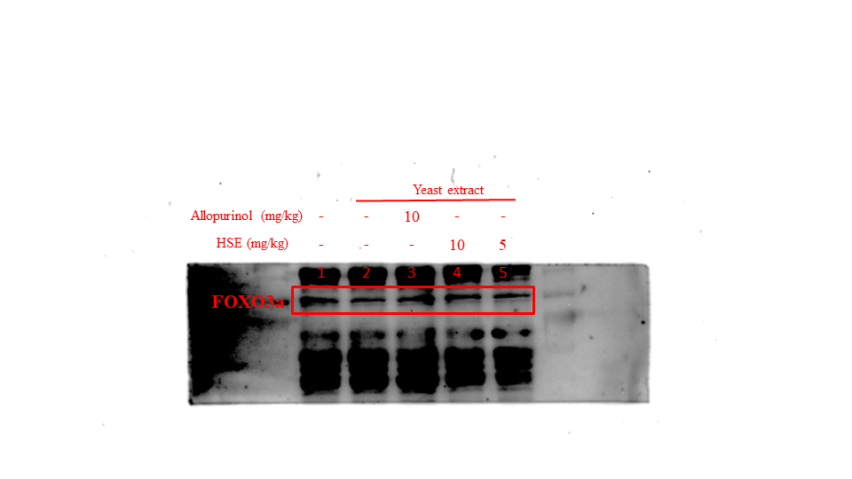
**
